# No association between variations in extracranial venous anatomy and clinical outcomes in multiple sclerosis patients over 5 years

**DOI:** 10.1186/s12883-019-1350-2

**Published:** 2019-06-11

**Authors:** Sirin Gandhi, Karen Marr, Marcello Mancini, Maria Grazia Caprio, Dejan Jakimovski, Avinash Chandra, Jesper Hagemeier, David Hojnacki, Channa Kolb, Bianca Weinstock-Guttman, Robert Zivadinov

**Affiliations:** 1 Buffalo Neuroimaging Analysis Center, Department of Neurology, Jacobs School of Medicine and Biomedical Sciences, University at Buffalo, State University of New York, Buffalo, NY USA; 2 Institute of Biostructure and Bioimaging, National Research Council of Italy, Rome, Italy; 3 Jacobs Multiple Sclerosis Center, Department of Neurology, School of Medicine and Biomedical Sciences, University at Buffalo, State University of New York, Buffalo, NY USA; 4 Center for Biomedical Imaging at Clinical Translational Science Institute, University at Buffalo, State University of New York, Buffalo, NY USA

**Keywords:** Multiple sclerosis, Extracranial, Variations in extracranial venous anatomy, CCSVI, Relapse rate, Disability progression, Longitudinal

## Abstract

**Background:**

No longitudinal, long-term, follow-up studies have explored the association between presence and severity of variations in extracranial venous anatomy, and clinical outcomes in patients with multiple sclerosis (MS).

**Objective:**

This prospective 5-year follow-up study assessed the relationship of variations in extracranial venous anatomy, indicative of chronic cerebrospinal venous insufficiency (CCSVI) on Doppler sonography, according to the International Society for Neurovascular Disease (ISNVD) proposed consensus criteria, with clinical outcomes and disease progression in MS patients.

**Methods:**

90 MS patients (52 relapsing-remitting, RRMS and 38 secondary-progressive, SPMS) and 38 age- and sex-matched HIs were prospectively followed for 5.5 years. Extracranial and transcranial Doppler-based venous hemodynamic assessment was conducted at baseline and follow-up to determine the extent of variations in extracranial venous anatomy. Change in Expanded Disability Status Scale (∆EDSS), development of disability progression (DP) and annualized relapse rate (ARR) were assessed.

**Results:**

No significant differences were observed in MS patients, based on their presence of variations in extracranial venous anatomy at baseline or at the follow-up, in ∆EDSS, development of DP or ARR. While more MS patients had ISNVD CCSVI criteria fulfilled at baseline compared to HIs (58% vs. 37%, *p* = 0.03), no differences were found at the 5-year follow-up (61% vs. 56%, *p* = 0.486).

**Discussion:**

This is the longest follow-up study assessing the longitudinal relationship between the presence of variations in extracranial venous anatomy and clinical outcomes in MS patients. *Conclusion:* The presence of variations in extracranial venous anatomy does not influence clinical outcomes over the 5-year follow-up in MS patients.

**Electronic supplementary material:**

The online version of this article (10.1186/s12883-019-1350-2) contains supplementary material, which is available to authorized users.

## Background

Multiple sclerosis (MS) is as an autoimmune disease affecting the central nervous system (CNS) that is characterized by demyelination and neurodegeneration from the earliest stages of its clinical presentation [1].

Compared with the arterial system, the development of the extracranial venous anatomy is subject to many variations. In the past, these variations were acknowledged as non-pathological findings Although the possible role of variations in extracranial venous anatomy in the development of the MS was first questioned more than one and a half centuries ago [2], and ever since, sparse cumulative evidence investigating the vascular etiology of MS pathogenesis has been documented over the years [3]. However, it was only recently that a causal association between variations in extracranial venous anatomy suggestive of chronic cerebrospinal venous insufficiency (CCSVI) and MS has been implied [4]. CCSVI is characterized by the variations in of main extracranial cerebrospinal venous outflow routes (obstructions of the internal jugular veins, IJV and/or azygos vein) that interfere with normal venous drainage, as evidenced by Doppler sonography (DS) [4, 5], although other non-invasive [6] and invasive [4, 7, 8] imaging methods were also used to detect these variations in extracranial venous anatomy. These variations in extracranial venous anatomy can be extra- or intra-luminal, and are may be a result of congenital truncular venous malformations, which represent an embryologically defective vein where developmental arrest has occurred during the vascular trunk formation period in the ‘later stage’ of the embryonic development [6].

Although the condition was originally described in MS patients, the results from independent controlled studies demonstrated that there is highly variable prevalence of this condition in patients with other CNS disorders and in healthy individuals (HI) [5, 8–13]. As more research studies became available, the concept of CCSVI as a pathologic condition, its diagnostic utility and clinical impact for MS patients have been questioned, given that no causal relationship between CCSVI and MS has been confirmed [6, 14]. A recent meta-analysis, including available studies investigating the association of CCSVI frequency with MS, reported considerable heterogeneity across the studies and a conservative sensitivity analysis yielded null association [14].

Although there is abundant cross-sectional data on this subject [6], there were only a few studies examining the association of CCSVI and clinical outcomes in MS patients over short-term [7, 15–19], while no long-term prospective studies have been reported, thus far. Therefore, the objective of this study was to explore whether there is an association between presence and severity of variations in extracranial venous anatomy, indicative of CCSVI, and clinical outcomes in patients with MS over 5 years.

## Methods

### Subjects

This 5-year follow-up is a part of an ongoing single-center, longitudinal, observational, case-control Cardiovascular, Environmental and Genetic risk factor in MS (CEG-MS) study that is recruiting subjects in Buffalo, NY [20]. The study was approved by the local Institutional Review Board of the University of Buffalo, USA and University of Naples, Italy, and written informed consent was obtained from each participant. The inclusion criteria for this follow-up CEG sub-study of variations in extracranial venous anatomy and clinical outcomes are: a) subjects originally enrolled in the CEG-MS study in years 2009 through 2013 [5, 20], b) age 18–75 years at baseline, and c) being MS patients or HI with unknown history of neurological disease. Exclusion criteria are: a) presence of relapse and steroid treatment within 30 days preceding their enrollment in the study (for MS patients), b) pre-existing medical conditions known to be associated with cerebral pathology (cerebrovascular disease, positive history of alcohol abuse), c) evidence of brain ischemic or hemorragic infarcts, or space-occupying lesions on MRI exam performed within 30 days of physical/neurologic examination with the standardized study protocol, and d) pregnancy. In total, 128 consecutive subjects consisting of 90 MS patients (52 with relapsing-remitting, RR and 38 with secondary-progressive, SP disease subtype) and 38 HI with unknown history of neurological disease were enrolled in this CEG-MS sub-study by November 2016, when the first database lock occurred. Seven MS patients enrolled in the study had undergone percutaneous venous angioplasty at external centers for the treatment of their CCSVI at independent time points unrelated to the current study.

### Clinical examination

MS was defined using the revised McDonald criteria [21] and all subjects were examined with physical and neurological examination by experienced neurologists, who were blinded to the results of the DS examination. Expanded Disability Severity Scale (EDSS) score was obtained for each MS patient at baseline and at follow-up. An absolute change in EDSS (ΔEDSS) was calculated, and development of disability progression (DP) was documented as a clinical measure of irreversible neurological disability. DP was defined as an absolute change in EDSS over 5 years with an increase in EDSS of ≥1.5 point if the baseline EDSS was 0, or ≥ 1.0 point if the baseline was between 1.0–5.5, or ≥ 0.5 point if the baseline EDSS was ≥6.0. The medical history and relapses were documented during the 5-year follow-up study assessment.

### Doppler sonography assessment

Doppler assessment was performed at baseline and at follow-up on all study participants using the same echo color Doppler machine, MyLab 25 Gold (Esaote-Biosound, Genoa, Italy) equipped with 2.5 MHz and 7.5–10 MHz transducers, and by a single Doppler sonographer who was blinded to the disease status of the subjects. The baseline blinding was achieved, by instructing subjects not to reveal their disease status during the examination, including patients with no disability or walking difficulties to ensure blinding between non-disabled MS patients and HIs [5]. At the follow-up, the blinding was achieved by positioning subjects supine in a tilt chair by a non-blinded personnel, draping from neck down and removing any assistive device used by the patients from the room before the Doppler sonographer entered, to avoid any visual cues of the presence of disease. The reproducibility of our approach was reported previously [5].

Detailed description of the baseline DS assessment of the extracranial (IJV and vertebral veins, VV) and intracranial venous system was previously reported [5]. Briefly, each subject was examined on a tilt chair in supine (0°) and sitting position (90°) allowing a minimum of 2 min post positional change for vascular flow redistribution prior to DS assessment. At the follow-up, the subjects were also examined on a tilt chair in supine and sitting position. All baseline and follow-up exams were examined in a blinded manner by a certified centralized reader (MM) at the University of Naples, Italy. The detection of extracranial venous variations in extracranial venous anatomy focused on the detection of 5 anomalous venous hemodynamic (VH) criteria [5], with presence of ≥2 VH criteria indicating the fulfillment of CCSVI criteria, according to the International Society for Neurovascular Disease (ISNVD) consensus statement [22]. Due to the technical discrepancy in the assessment of the VH criteria at baseline and at the follow-up, the VH criteria 1 and 2 were not comparable between the two time-points [22].

### Statistical analyses

Statistical analysis was performed using the IBM Statistical Package for the Social Sciences (SPSS version 24.0). Student t-test, chi-square test and Mann Whitney U-test were used testing the differences between study groups, as appropriate. Wilcoxon signed-rank test was used to compare the individual VH criteria within the study groups between the baseline and the follow-up. As there was no significant difference observed in age, gender or disease duration between the study groups (MS vs HIs), chi-square test or Wilcoxon signed-rank test was used to investigate the association between the CCSVI status or the individual venous hemodynamic criteria. The evolution of clinical outcome measures (annualized relapse rate, ARR, ∆EDSS and DP) were analyzed using independent-sample t-test, Mann Whitney U-test and chi-square test as appropriate. A nominal *p*-value of ≤0.05 was considered statistically significant, and *p* < 0.1 was considered a trend, using two-tailed test.

## Results

### Demographic and clinical characteristics

The demographics and clinical characteristics of the study subjects are presented in Table 1. The mean age at baseline was 45.1 years in HIs and 47.3 years in MS patients (*p* = 0.387). The average follow-up duration was similar in MS patients and HIs (*p* = 0.342). No significant differences were observed in the confounding variables between the study groups in terms of their gender, body mass index (BMI), prevalence of pre-existing comorbidities such as hypertension, hyperlipidemia or diabetes mellitus.

**Table 1:** Demographic and clinical characteristics of the study participants

	HI (*n* = 38)	MS (*n* = 90)	*p* value
Female, n (%)	25 (67)	66 (73)	0.387
Baseline age (yrs); mean (SD)	45.1 (13.6)	47.3 (10.4)	0.309
Time to F/up (yrs); mean (SD)	5.4 (0.3)	5.5 (0.5)	0.342
Baseline BMI (kg/m^2^); mean (SD)	26.6 (5.8)	27.7 (5.9)	0.363
Follow-up BMI (kg/m^2^); mean (SD)	26.2 (5.2)	27.9 (5.9)	0.363
Baseline comorbidities, n (%)			
Hypertension	6 (16)	11 (12)	0.586
Hyperlipidemia	6 (16)	11 (12)	0.586
Diabetes	2 (5)	2 (4)	0.372
Follow-up comorbidities, n (%)			
Hypertension	9 (24)	15 (17)	0.353
Hyperlipidemia	10 (26)	22 (23)	0.719
Diabetes	3 (8)	4 (4)	0.433
Baseline disease duration (yrs), mean (SD)	N/A	15.3 (10.0)	N/A
Disease progression, n (%)			
CIS → RR	N/A	7 (8)	N/A
RR → SP		11 (13)	
Remained RR		46 (51)	
SP since baseline		27 (30)	
Baseline EDSS, median (IQR)	N/A	3.0 (1.5–5.5)	N/A
F/up EDSS, median (IQR)	N/A	3.5 (2.0–6.0)	N/A
∆EDSS, mean (SD); median	N/A	0.3 (0.9); 0.5	N/A
DP at F/up, n (%)	N/A	25 (28)	N/A
Annualized relapse rate at F/up, mean (SD)	N/A	0.2 (0.4)	N/A
Relapse free from baseline to follow-up, n (%)	N/A	55 (61)	N/A
Baseline DMT status, n (%)			
Interferon-beta 1a	N/A	34 (38)	N/A
Glatiramer acetate		20 (22)	
Natalizumab		18 (20)	
Other DMT*		4 (4)	
No DMT		14 (16)	
Follow-up DMT status, n (%)			
Remained on same DMT	N/A	47 (52)	N/A
Switched to another DMT		33 (37)	
No DMT		10 (11)	

*HI* Healthy individual, *MS* multiple sclerosis, *n* number, *SD* standard deviation, *BMI* Body Mass Index, *yrs* Years, *F/up* Follow-up, *CIS* Clinically isolated syndrome, *RR* relapsing-remitting, *SP* secondary-progressive, *DP* disability progression, *EDSS* Expanded Disability Status Scale, *∆EDSS* Absolute change in EDSS, *IQR* interquartile range, *DMT* disease-modifying treatment

All p-values were calculated using independent-sample t-test, Mann Whitney U-test and chi-square test as appropriate

*Other DMTs include intravenous immunoglobulin, mitoxantrone and azathioprine

In the MS group, 7 (8%) of the patients had progressed from clinically isolated syndrome (CIS) to relapsing-remitting (RR) MS disease subtype, and 10 (11%) from RRMS to secondary-progressive (SP) MS (Table 1). The DP was observed in 25 (28%) of the patient population (Table 1). Eighty (89%) of MS patients were on disease-modifying treatment at the time of their follow-up visit (Table 1). Median ΔEDSS over the follow-up was 0.5, mean annualized ARR was 0.2 per year, and 61% of the patients remained relapse-free from baseline to follow-up.

Detailed demographic and clinical characteristics of the RRMS and SPMS patients at baseline are provided in the Additional file 1: Table S1. Demographic data was also compared between subjects fulfilling and not-fulfilling ISNVD CCSVI criteria at baseline, to understand the influence of these variables on the outcome measures over the follow-up (Table 2). No significant differences were observed between these groups in terms of age, disease duration, BMI, comorbidities or follow-up duration.

**Table 2:** Demographic and clinical characteristics of the multiple sclerosis patients, according to their fulfillment of CCSVI criteria at baseline

	CCSVI ISNVD criteria fulfilled [22] (*n* = 52)	CCSVI ISNVD criteria not-fulfilled [22] (*n* = 38)	*p* value
Female, n (%)	37 (71)	29 (76)	0.584
Baseline age (yrs); mean (SD)	47.6 (10.7)	47 (10.0)	0.815
Time to F/up (yrs); mean (SD)	5.5 (0.5)	5.5 (0.4)	0.810
Baseline BMI (kg/m^2^); mean (SD)	27.3 (5.4)	28.7 (6.5)	0.996
Follow-up BMI (kg/m^2^); mean (SD)	29.1 (4.9)	27.2 (6.5)	0.212
Baseline comorbidities, n (%)			
Hypertension	4 (8)	7 (18)	0.125
Hyperlipidemia	6 (12)	5 (13)	0.817
Diabetes	1 (2)	1 (3)	0.822
Follow-up comorbidities, n (%)			
Hypertension	7 (13)	8 (22)	0.075
Hyperlipidemia	13 (24)	8 (22)	0.644
Diabetes	2 (4)	2 (6)	0.652
Baseline disease duration (yr), mean (SD)	15.0 (10.2)	14.4 (9.5)	0.747
Disease progression, n (%)			
CIS → RR	3 (6)	4 (11)	N/A
RR → SP	7 (14)	4 (11)	
Remained RR	25 (48)	21 (55)	
SP since baseline	17 (33)	9 (24)	
Baseline DMT status, n (%)			
Interferon-beta 1a	17 (33)	17 (48)	N/A
Glatiramer acetate	13 (25)	7 (18)	
Natalizumab	13 (25)	5 (13)	
Other DMT*	1 (2)	2 (5)	
No DMT	8 (15)	6 (16)	
Follow-up DMT status, n (%)			
Remained on same DMT	27 (52)	22 (58)	N/A
Switched to another DMT	17 (33)	13 (34)	
No DMT	8 (15)	3 (8)	

*n* number, *SD* standard deviation, *BMI* Body Mass Index, *yr* Years, *F/up* Follow-up, *CIS* Clinically isolated syndrome, *RR* relapsing-remitting, *SP* secondary-progressive, *DMT* disease-modifying treatment ISNVD-International Society for Neurovascular disease

All *p*-values were calculated using independent-sample t-test, Mann Whitney U-test and chi-square test as appropriate

*Other DMTs include intravenous immunoglobulin, mitoxantrone and azathioprine

### Venous hemodynamic differences between and within study groups

Table 3 shows individual VH criteria and frequency of subjects fulfilling ISNVD CCSVI criteria in HIs and MS patients at baseline, and at the follow-up. At baseline, 52 (58%) of MS patients and 14 (37%) of HIs (*p* = 0.03) fulfilled ISNVD CCSVI criteria. At the follow-up, the ISNVD CCSVI criteria were fulfilled in 55 (61%) of MS patients and 21 (56%) of HIs (*p* = 0.486).

**Table 3:** Comparison of venous hemodynamic Doppler sonography criteria at baseline and at follow-up study visit in healthy individuals and multiple sclerosis patients

VH Criteria at Baseline	HI (n=38)	MS (n = 90)	*p*-value
Criterion 1 positivity, n (%) *- IJV and/or VV reflux*	8 (21)	32 (36)	0.09
Criterion 2 positivity, n (%) - *Bidirectional IC venous outflow*	10 (26)	29 (32)	0.459
Criterion 3 positivity, n (%) - *B- mode IJV stenosis*	14 (37)	54 (60)	**0.011**
Criterion 4 positivity, n (%) - *No flow in IJV and/or VV*	3 (8)	10 (11)	0.557
Criterion 5 positivity, n (%) - *IJV CSA90° > IJV CSA0°*	1 (3)	6 (7)	0.346
CCSVI criteria fulfilled according to the ISNVD consensus [22], n (%)	14 (37)	52 (58)	**0.03**
**VH Criteria at the follow-up**	**HI (n=38)**	**MS (n = 90)**	***p*** **-value**
Criterion 1 positivity, n (%) *- IJV and/or VV reflux*	0 (0)	3 (3)	0.255
Criterion 2 positivity, n (%) - *Bidirectional IC venous outflow*	29 (32)	77 (86)	0.206
Criterion 3 positivity, n (%) - *B- mode IJV stenosis*	26 (68)	55 (61)	0.433
Criterion 4 positivity, n (%) - *No flow in IJV and/or VV*	7 (18)	17 (19)	0.951
Criterion 5 positivity, n (%) - *IJV CSA90° > IJV CSA0°*	0 (0)	12 (13)	**0.018**
CCSVI criteria fulfilled according to the ISNVD consensus [22], n (%)	21 (56)	55 (61)	0.486

*CCSVI* Chronic cerebrospinal venous insufficiency, *n* number, *VH* Venous hemodynamic, *HI* Healthy individual, *MS* Multiple sclerosis, *IJV* Internal jugular vein, *VV* Vertebral vein, *IC* intra-cranial, *CSA* Cross sectional area, *ISNVD* International Society for Neurovascular Disease

All p-values were calculated using Chi square and Fisher’s exact test, and *p* values <0.05 were considered significantt

At the baseline visit, there was a significantly higher prevalence of positive VH criterion 3 in MS patients compared to HIs (54 vs 14, *p* = 0.011). At the 5-year follow-up visit, there was a significantly higher prevalence of positive VH criterion 5 in MS patients compared to HIs (13 vs 0, *p* = 0.018), as none of the HIs presented with negative ΔCSA.

Wilcoxon signed-rank test showed no within group changes in VH criteria positivity or the fulfillment of the ISNVD CCSVI criteria in MS patients and HIs between baseline and the follow-up. A trend toward significance was observed in VH criterion 5 positivity in MS patients from baseline to follow-up (Z = − 1.732; *p* = 0.083). On further stratification of MS patients, 9 (24%) of SPMS had positive VH criterion 5 at the follow-up, which was significantly greater compared to baseline (Z = − 2.33; *p* = 0.02).

### *Evolution of clinical outcomes according to the baseline* fulfillment of the ISNVD CCSVI criteria

Table 4 shows evolution of clinical outcomes, according to the baseline fulfillment of the ISNVD CCSVI criteria. At baseline, there was a trend for higher EDSS in those who fulfilled, compared to those who did not fulfill the criteria (*p* = 0.08), which was not evident at the follow-up (*p* = 0.11). No differences between those who fulfilled ISNVD CCSVI criteria and those who did not were observed over the follow-up in ΔEDSS, development of DP, number of the relapses between baseline and follow-up, ARR or being relapse-free. No differences in evolution of clinical outcomes was found, according to the baseline fulfillment of the ISNVD CCSVI criteria when RRMS and SPMS patients were considered separately.

**Table 4:** Evolution of clinical outcomes over 5 years in multiple sclerosis patients, according to their baseline CCSVI status

	Total (n = 90)	CCSVI CCSVI ISNVD criteria fulfilled [22] (*n* = 52)	CCSVI CCSVI ISNVD criteria not-fulfilled [22] (n = 38)	*p* value
EDSS at baseline, median (IQR)	3.0 (1.5–5.5)	3.5 (2.0–6.0)	2.5 (1.5–4.0)	0.08
EDSS at follow-up, median (IQR)	3.5 (2.0–6.0)	3.5 (1.7–6.5)	3.0 (2.0–4.0)	0.11
∆EDSS, mean (SD); median	0.3 (0.9); 0.5	0.4 (0.7); 0.5	0.2 (1.0); 0.3	0.76
DP at follow-up, n (%)	25 (28)	16 (31)	9 (24)	0.50
Number of relapses between baseline and follow-up, mean (SD)	0.9 (2.0)	0.8 (1.6)	1.0 (2.5)	0.52
Annual relapse rate over the follow-up, mean (SD)	0.2 (0.4)	0.1 (0.3)	0.2 (0.5)	0.52
Relapse free from baseline to follow-up, n (%)	55 (61)	33 (64)	22 (58)	0.60

*CCSVI* Chronic cerebrospinal venous insufficiency, *EDSS* Expanded Disability Status Scale, *∆EDSS* Absolute change in EDSS from baseline to follow-up, *IQR* interquartile range, *SD* standard deviation, *n* number, *DP* Disability progression, *ISNVD* International Society for Neurovascular Disease

All p-values were calculated using independent-sample t-test, Mann Whitney U-test and chi-square test as appropriate

### Clinical outcomes of MS patients who underwent percutaneous venous angioplasty

Of 7 MS patients who underwent percutaneous angioplasty, 3 (42.9%) were in RRMS and 4 (57.1%) in the SPMS group. Six (86%) of those fulfilled ISNVD CCSVI criteria at baseline and their median EDSS was 4.0. Median time period from the baseline to the date of procedure was 3 months. At their follow-up, 57% patients continued to have fulfilled ISNVD CCSVI criteria. The mean ∆EDSS was 0.2 in RRMS and 0.8 in SPMS patients, respectively over the follow-up. DP was detected in 2 of the 7 patients (33%) and the mean ARR of this subgroup was 0.13.

## Discussion

To the best of our knowledge, this is the longest follow-up study assessing the longitudinal relationship between the presence of variations in extracranial venous anatomy and clinical outcomes in MS patients. The association of variations in extracranial venous anatomy with disability status in MS patients has been a debatable topic over the last decade. Our study found that the presence of variations in extracranial venous anatomy is not exclusive to the MS patients, as there was no significant difference in the prevalence of those in MS patients versus HIs, at the follow-up. Thus, a causal association between the two cannot be implied. Furthermore, a very small subset of our study population underwent venous angioplasty which did not alter the disease course in these patients. Due to the small sample size, these results are only descriptive and no statistical tests have been performed on these data.

The heterogeneity of the extracranial and intracranial venous system was documented using various imaging modalities including DS, MR or contrast venography [6]. The capacitance of the extracranial neck vessels is highly sensitive to alteration in patient position, hydration status and use of imaging methodology, thus, making standardized assessment challenging, especially longitudinally and particularly using DS [6]. These practical limitations should be kept in mind, when interpreting results of this follow-up study.

With total of 20% of the patients progressing from CIS to RR or from RR to SP MS, and DP occurring in 28% of the patients, this marks the first case-control study so far, to investigate the impact of variations in extracranial venous anatomy on disease progression in MS patients and its subtypes (relapsing vs progressive). At baseline, we found that prevalence of variations in extracranial venous anatomy was significantly higher in MS patients compared to HIs. However, no differences between MS patients and HIs was found at the follow-up.

The analysis of the current study has concluded on similar lines as our previously reported post hoc *analysis* of the cross-sectional CEG-MS study and other reports, which failed to document an association between CCSVI severity and clinical outcomes in MS patients [13, 23]. Although at baseline, there was a trend towards higher EDSS in patients fulfilling ISNVD CCSVI criteria, this pattern did not continue during our follow-up. In addition, we did not find apparent association between the baseline presence or severity of variations in extracranial venous anatomy and development of DP or change in ∆EDSS, indicating no predictive value in monitoring disability progression. This finding stresses the importance of conducting prospective studies when exploring associations between different conditions.

An interesting finding in our SPMS subgroup was that these patients demonstrated a significant lack of posture-dependent IJV collapse (measured using negative IJV cross-sectional area, as part of the VH criterion 5), while a trend towards significance was observed similarly in the overall MS group. IJV collapse in orthostatism is a physiologic phenomenon influenced by decreased hydrostatic pressure [24]. To better understand the hemodynamic implications of the above, simultaneous dynamic assessment of cerebral venous outflow should be incorporated. Recent quantitative study of cerebral blood flow has implied an association between altered postural regulation of inflow-outflow in the cerebral vasculature of MS patients [25].

A concerning aspect of the hypothesis of etiopathogenic role of CCSVI in MS was the introduction of an interventional procedure to correct the deformity [26]. Use of percutaneous venous angioplasty has been complicated by serious adverse events such as fatal brainstem hemorrhage, iatrogenic cerebral venous thrombosis, stent migration and in-stent thrombosis, among others [27]. The PREMiSE, a double-blinded randomized trial for the use of venous angioplasty in MS patients demonstrated no benefit in clinical or radiological outcomes with use of venous angioplasty [7]. Another randomized, double-blind, sham-controlled trial, Brave Dreams, has shown no clinical or radiological benefit of venous angioplasty for CCSVI in patients with MS [28]. Though, only 8% of our MS subset had undergone this procedure at outside facilities in the present study, no improvement in clinical outcomes was seen in our patients despite treatment for the underlying variations in extracranial venous anatomy.

The strengths of the present study include a prospective study design with a long follow-up duration of 5.5 years, the employment of blinding Doppler sonographer and neurologists to minimize the ascertainment bias between the study groups, as well as the use of blinded centralized reading of the baseline and follow-up Doppler examinations. All clinical outcome measures and Doppler parameters were assessed at the follow-up by the same personnel, as at baseline to avoid inter-rater variability.

However, a major limitation of this study was that there was a change in technical assessment of VH criteria 1 and 2 [22], which limited their use for comparison between the two studies timepoints. This further emphasizes that use of DS is not an adequate approach for monitoring variations in extracranial venous anatomy in both cross-sectional and longitudinal studies. Moreover, a number of recent multimodal diagnostic studies showed inadequate sensitivity and specificity of DS in screening real frequency of variations in extracranial venous anatomy [7, 8, 29], which further outlines a need of applying multimodal approach to screen and monitor these variations in extracranial venous anatomy by using more accurate invasive and non-invasive imaging techniques [8, 22, 30]. Furthermore, Type II error may be a possibility due to limited sample size. However, we had an 80% power to detect a medium (0.60) effect (G*Power 3.1.9.2), suggesting the study was neither over- nor under-powered. Taken together with the highly similar 5-year change in the clinical outcomes between groups (*p* ≥ 0.52), false negative are unlikely.

## Conclusions

In conclusion, our findings showed that the presence of variations in extracranial venous anatomy was not associated with disease progression or increased clinical activity in MS patients over the 5-year follow-up.

## Additional file

Additional file 1: **Table S1.** Demographics and clinical characteristics in multiple sclerosis patients, according to their disease subtype

## Data Availability

Availability of data and materials will be made available on a reasonable request.
