# Supplementary material for: No association between variations in extracranial venous anatomy and clinical outcomes in multiple sclerosis patients over 5 years
Source: BMC Neurol. 2019 Jun 11;19:121. doi: 10.1186/s12883-019-1350-2 (PMC6560860; doi:10.1186/s12883-019-1350-2)
Supplement: Supplementary file 1 — Table S1. Demographics and clinical characteristics in multiple sclerosis patients, according to their disease subtype [file 12883_2019_1350_MOESM1_ESM.docx]

*Additional file 1.*

File name: Supplement Table

|  | **MS Subtypes** | | **P-value** |
| --- | --- | --- | --- |
|  | **RRMS**  **(n=52)** | **SPMS**  **(n=38)** |  |
| Female, n (%) | 36 (68) | 30 (79) | 0.30 |
| Baseline age (yrs); mean (SD) | 42.8 (9.4) | 53.6 (8.2) | **<0.001** |
| Time to F/up (yrs); mean (SD) | 5.5 (0.5) | 5.5 (0.4) | 0.88 |
| Baseline disease duration (yr), mean (SD) | 10.5 (7.5) | 20.8 (9.8) | **<0.001** |
| Baseline EDSS, median (IQR) | 2.0 (1.5-2.5) | 5.0 (3.5-6.5) | **<0.001** |
| F/up EDSS, median (IQR) | 2.3 (1.5-3.0) | 6.0 (4.0-6.5) | **<0.001** |
| ∆EDSS, mean (SD); median | 0.4 (1.0);0.5 | 0.2 (0.7);0.0 | 0.22 |
| DP, n (%) | 12 (23) | 13 (34) | 0.49 |
| Annual relapse rate at F/up, mean (SD) | 0.2 (0.4) | 0.1 (0.3) | 0.33 |
| Relapse free from baseline to follow-up, n (%) | 27 (52) | 28 (74) | **0.04** |
| Baseline DMT group, n (%)  Interferon-beta 1a  Glatiramer acetate  Natalizumab  Other DMT*  No DMT | 18 (34)  11 (21)  13 (25)  1 (2)  9 (16) | 17 (46)  9 (24)  5 (14)  2 (5)  5 (13) | 0.77 |
| Follow- up treatment, n (%)  Remained on same DMT  Switched to another DMT  No DMT | 28 (53)  19 (36)  5 (10) | 19 (51)  13 (35)  6 (16) | 0.70 |

**Table S1.**  Demographics and clinical characteristics in multiple sclerosis patients, according to their disease subtype.

**Legend:** MS – multiple sclerosis; n – number; SD – standard deviation; BMI – Body Mass Index; yrs- Years; F/up- Follow-up; CIS- Clinically isolated syndrome; RR – relapsing-remitting; SP – secondary-progressive; DP –disability progression; EDSS – Expanded Disability Status Scale; ∆EDSS- Absolute change in EDSS; IQR – interquartile range; yr – years; DMT – disease-modifying treatment; All p-values calculated using independent-sample t-test, Mann Whitney U-test and chi-square test as appropriate

*- Other DMTs include intravenous immunoglobulin, mitoxantrone and azathioprine.
